# “Doctors Aren’t Familiar with Your Tissues”: Self-Examination and Feminist Health Activism in 1970s Canada

**DOI:** 10.3138/chr-2022-0032

**Published:** 2023-06

**Authors:** Karissa Patton, Whitney Wood

**Affiliations:** University of Edinburgh, Edinburgh, Scotland; Vancouver Island University, Nanaimo, British Columbia, Canada

**Keywords:** self-examination, women’s health, feminist activism, alternative health models, health education, prevention, gynecology, medical authority, auto-examen, santé des femmes, activisme féministe, modèles de santé parallèles, éducation à la santé, prévention, gynécologie, autorité médicale

## Abstract

This article explores the impact of self-examination as a tool of feminist resistance, an act of preventive health care, and a site where mainstream and alternative health models were debated in late twentieth-century Canada. In the early 1970s, a range of women’s health groups increasingly turned their liberationist critiques towards the structures of mainstream medicine, and the self-exam became a vehicle that allowed women to push back against what they cast as the systemic power imbalances involved with the traditional doctor-patient relationship. Both breast and pelvic self-exams became staples of the women’s health movement as feminists encouraged women to take health care into their own hands, both figuratively and literally. As the decade progressed, breast self-examination transformed from a niche feminist technique to a relatively commonplace preventative health practice, increasingly discussed within popular women’s magazines across North America. Pelvic self-examination remained more controversial as the practice was denounced by a small but vocal group of Canadian physicians, resentful of lay incursions into medical practice. Drawing on women’s magazines and feminist newsletters, archival files from Canadian women’s health centres, and debates about self-examination in national newspapers, we reveal how shifting narratives about women’s liberation, responsibility for preventative health practices, and medical authority intersected in the feminist practice of self-examination.

## Résumé

Le présent article examine les efets de l’auto-examen en tant qu’outil de résistance féministe, acte de soins de santé préventifs et lieu de débat sur les modèles de santé conventionnels et parallèles dans le Canada de la fin du xxe siècle. Au début des années 1970, divers groupes de défense de la santé des femmes ont de plus en plus dirigé leurs critiques libérationnistes vers les structures de la médecine traditionnelle; l’auto-examen a quant à lui permis aux femmes de s’opposer à ce qu’elles considéraient comme des déséquilibres de pouvoir systémiques liés à la relation traditionnelle entre le médecin et sa patiente. Les auto-examens mammaire et pelvien se sont révélés des éléments essentiels du mouvement pour la santé des femmes, car les féministes encourageaient les femmes à prendre leur santé en main, au sens figuré comme au sens propre. Au cours de la décennie, une transformation s’est opérée : de technique féministe qu’il était, l’auto-examen des seins est devenu une pratique de santé préventive relativement banale, dont traitaient de plus en plus les magazines féminins populaires d’Amérique du Nord. L’auto-examen pelvien est resté plus controversé, car il a été dénoncé par un petit groupe de médecins canadiens, qui ne cachaient pas leur ressentiment à l’égard des incursions de profanes dans la pratique médicale. En s’appuyant sur les magazines féminins et les bulletins féministes, les dossiers d’archives des centres de santé des femmes et les débats sur l’auto-examen dans les journaux canadiens, les auteures montrent l’entrecroisement et l’évolution du discours sur la libération de la femme, la responsabilité pour les pratiques de santé préventives et l’autorité médicale dans la pratique féministe de l’auto-examen.

## Introduction

In November 1977, Vancouver-based journalist Anne Roberts published a piece in the *Globe and Mail*, highlighting the work of the Vancouver Women’s Health Collective (vwhc), a group formally incorporated in 1972.^1^ Roberts opened the piece: “A fushed face, swollen neck glands, irritated skin – each symptom is a familiar, easily detected change in the body that most people can diagnose correctly as a cold setting in, the beginnings of the flu, or an allergic reaction to a cat. But some equally obvious changes that take place in a woman’s breasts or cervix could be signs that cancer has struck, go unnoticed or are ignored because women don’t know what they mean.”^2^ Describing the collective’s work to redress women’s long-standing unfamiliarity with their bodies – particularly those areas that, in her words, had “long been considered ‘private parts’” – Roberts detailed the vwhc’s efforts to promote breast and cervical self-examination through regular Tuesday evening clinics. Noting the value of self-examination in understanding one’s own body and health, particularly when it came to breast cancer (“the number one killer of women between 30 and 60” and a disease for which, the piece rightly pointed out, early detection was key), the article quoted vwhc member “Jackie” who commented: “The best way to find tumours is to do your own self-examination. Doctors aren’t familiar with your tissues. They can’t remember you from one year to the next.”^3^ Feminist health organizations like the vwhc sought to teach women that they “have the right to see, to touch, and to care for their own bodies,” and self-examination was positioned as an important part of preventative health care.^4^ Encouraged to know and to monitor their own anatomy, women established an individualized personal baseline of breast and pelvic health that had the potential to valuably inform their interactions with physicians and other health practitioners.^5^

A growing part of feminist health activism throughout North America from the early 1970s onwards, self-examination techniques, including both breast self-exam (bse) and cervical self-exam (cse), attracted attention in the mainstream Canadian press by the end of the decade. Drawing on a multi-sited analysis of feminist publications and the archived materials of women’s health collectives, leading women’s magazines published in Canada and the United States, and popular Canadian newspapers, this article explores the ways in which self-examination was positioned by English Canadian feminists as an essential tool of health education, preventative care, and health activism during the 1970s. Focusing, additionally, on the ways in which feminist health activists, social and cultural commentators, and health practitioners linked and distinguished between the two sites of self-examination – breast and pelvic – in activist and popular discourses, we also explore growing pushback against feminist efforts to use the self-exam as a tool that figuratively and literally placed “women’s health in women’s hands” in the late 1970s and 1980s. Ultimately, the self-examination functioned as a key site where these complex tensions between mainstream and alternative medical models, and medical professionals and lay practitioners, played out during this transformative period of women’s liberation and Canadian Medicare policy.

Focusing on the individual and collective experiences of those who engaged in the self-examination of reproductive organs classified as “female,” we wish to offer a brief note on the language used throughout the article: we apply the terms “woman” and “women” to refer to those who practised and engaged in activism around self-exams during this period when appropriate to reflect the language used in the primary source materials that are discussed. Wherever possible, we seek to move beyond biologically essentialist descriptors and adopt language to centre the health experiences of all those who identify as women, non-binary, or gender non-conforming. In so doing, we aim to move towards a more inclusive approach to studying the history of self-examination in relation to the broader history of “women’s health.”

## Situating the Self-Exam in Histories of Women’s Health and Canadian Health Care

The history of “women’s health” emerged at the intersection of social history, the history of medicine, and women’s history in the 1970s. Early scholars working in the field understandably assigned pregnancy and childbirth a central place in their work and, building on these foundational efforts, researchers turned their attention to histories of birth control and abortion.^6^ Histories of women’s health in Canada have largely followed these trends, exploring representations and experiences of pregnancy, childbirth and motherhood, and contraception and abortion.^7^ In recent decades, scholars focusing on North America have considerably expanded the scope of the field, with new histories of gynecology building on germinal works including Wendy Mitchinson’s *The Nature of Their Bodies* and *Body Failure*.^8^ Historians including Patricia Jasen, Mandy Hadenko, and Jennifer Fraser, in particular, have made significant contributions to scholarly understandings of the metaphors and screening campaigns that surrounded conditions including breast, ovarian, and cervical cancer.^9^

Seeking to build on this historiography, this article engages with a rich body of work on feminist health activism, both in Canada and transnationally. Scholars including Susan Smith, Sandra Morgen, Wendy Kline, Jennifer Nelson, and Hannah Dudley-Shotwell have laid the foundation in exploring the history of women’s health activism during feminism’s so-called “second wave,” focusing on the United States,^10^ and they, along with Kathy Davis in her analysis of the global impact of *Our Bodies, Ourselves*, have aptly demonstrated “how feminism travels across borders.”^11^ Canadians regularly liaised with, and learned from, American health workers and self-help advocates who were developing transnational networks, and we are particularly indebted to this scholarship.

North of the border, Canadians engaged in grassroots feminist organizing around a number of health issues, ranging from birth reform to fat activism, during the 1970s.^12^ In weaving the history of self-examination into the broader Canadian landscape of feminist health activism, we build on foundational work from Christabelle Sethna and Megan Blair, who have demonstrated strong currents of reproductive rights activism among university students in Toronto, Montreal, and Waterloo, and have positioned campus women’s centres as important sites of feminist health care.^13^ We also draw on recent efforts to record individual stories of the women’s health movement.^14^ These existing feminist health histories, by and large, however, have tended to focus on cities in central Canada. Taking up the arguments of historians of medicine including Megan Davies, Catherine Carstairs, Esyllt Jones, and Lianne McTavish who have demonstrated the importance of fully accounting for regional diference, particularly in the Canadian health-care context,^15^ we aim to complement this work by focusing on histories of feminist health activism in two Western Canadian cities (as well as the surrounding rural communities they served, as was particularly the case in the prairie provinces), situating these case studies in the broader English Canadian context as much as possible.

This article focuses primarily on the local case studies of two women’s health centres established at the start of what we, alongside other social and cultural historians, position as the long 1970s,^16^ a key period in the history of feminist health activism: the Calgary Birth Control Association (cbca) and the Vancouver Women’s Health Collective (vwhc). The cbca was established in Calgary, Alberta, in 1970. While “birth control” was part of its name, the association provided services for a variety of reproductive and sexual health topics and needs.^17^ The vwhc was formally established in Vancouver, British Columbia, in 1972 and has had a long history of providing women’s health services and education, which continues to this day.^18^ Due to its devotion to collaborative knowledge production and sharing, the collective quickly became one of the most well-known women’s health organizations in Canada; the vwhc’s influence is seen in many other local case studies of women’s health centres in Canada and beyond, including many connections with the cbca.^19^

Examining local perspectives is essential to understanding how health care operated on a policy level and how people accessed services on the ground in the 1970s and 1980s. Case studies of the cbca and the vwhc offer invaluable insight into the broader context of health-care experiences and feminist health movements in Canada in the 1970s. While the federal government created a medical care insurance program that implemented cost sharing with the provinces and territories in 1966, the program did not offcially launch until 1968, and only two provinces immediately qualified at that time. The provincial governments of British Columbia and Alberta implemented their health-care policies under the cost-sharing program in 1968 and 1969 respectively, which did not include comprehensive policies for abortion and contraceptive services.^20^ The overlapped timing of the federal decriminalization of contraception and abortion in 1969 and the offcial implementation of provincial health care under the Canadian Medicare cost-sharing programs meant that many reproductive and sexual health services in each province remained in a bureaucratic limbo well into the 1970s and 1980s, and these policy bottlenecks fuelled the growth of feminist health activism and service provision at the local, regional, and provincial levels.^21^ Furthermore, as each province implemented its own unique health-care policy, the uptake of reproductive and sexual health services varied from province to province.^22^ The local stories of the cbca and the vwhc illustrate how women’s health centres established alternative health models, filled gaps in health services in the communities they served, and became critical parts of their respective local and provincial health-care landscapes in the 1970s and 1980s.

## Feminist Health Collectives, Self-Education, and Self-Help

cbca and vwhc workers and volunteers offered services that filled health-care needs in their communities and spearheaded initiatives that reffected their liberationist approach to health. As historians have demonstrated, shared “doctor stories” and negative encounters with medicine – experiences regularly shared in women’s health centres – fuelled emergent feminist and collective alternative health models in the late twentieth century.^23^ Throughout the 1970s and 1980s, cbca and vwhc educators provided opportunities for women to learn about individual and collective health experiences and their own bodies and to reclaim power over their own health. It is important to note, as Wendy Kline, Benita Roth, and Linda Nicholson have shown, that, while the women running these health centres hoped that all women could achieve self-empowerment and liberation, the concept that women shared a universal experience, central to what has been traditionally referred to as “second-wave” feminism, often privileged white, heterosexual, and middle-class perspectives.^24^

Both the vwhc and the cbca served clients from many racial, ethnic, and class backgrounds during the 1970s and sought community input in the development of their services. The volunteers and workers running these centres – in Vancouver, Calgary, and elsewhere – were similarly diverse in age and class, some growing up in these cities, while others had moved from smaller towns or rural communities but were mostly white.^25^ Despite their diferent backgrounds, as part of the women’s health movement more broadly at this time, those running these centres struggled with limited concepts of universal womanhood and the collective issues faced by women and those with bodies historically defned as “female.”^26^ Many in the women’s health movement, additionally, sought to embrace the idea of shared biological realities, like menstruation and menopause, as amplifying and solidifying universally “female” experiences. This emphasis on universalism shaped the goals of national and international women’s movements, othering and marginalizing the various realities of women of colour, Indigenous women, working-class women, and lesbians as niche issues. While a diverse clientele visited these women’s health spaces and utilized feminist health and self-help services in the 1970s, it was not until the late 1980s and 1990s that the specifc needs of women of colour and lesbians were taken up by many women’s health organizations.^^27^^

The concept of grassroots self-help was an integral part of women’s liberation praxis in the 1970s and was taken up in women’s health centres as a tool to reclaim power from the medical establishment. In 1972, the cbca joined forces with several other women’s liberation groups in Calgary to host the Self-Help Conference. The conference featured a number of panels and workshops that taught women various skills, including a popular panel on the “diy divorce.”^28^ The Calgary Self-Help Conference is just one example of how feminists across North America developed resources on legal, social, and health matters that encouraged women not to rely on men, personally or professionally, and to reclaim knowledge about their own rights and bodies. In the early 1970s, the vwhc created a pamphlet titled *The Concept of Self-Help* and positioned self-help as a proactive solution to the misogyny that many saw in the medical establishment: “As women we have had a lot of power over our own bodies and our health care taken away from us. One way in which women have begun to take back control is to collect and share information with each other. In fact, we all have valuable information and can share and learn it together. This concept is called ‘self-help.’”^^29^^

Local women’s health centres encouraged individuals to take their health into their own hands and tip the balance of power away from medical professionals, and, as a result, collective knowledge production and knowledge sharing became significant tools in attempts to foster women’s liberation in both the medical and social spheres. The creation and distribution of self-education materials on bodies, sex, and health was a large part of the education services that the cbca and vwhc provided throughout the 1970s and 1980s.^30^ M.J. Sobnosky argues that the self-help resources that these groups created “the substance out of which much of the [women’s health] movement’s rhetoric was formed.”^31^ Both the cbca and the vwhc housed large libraries and developed a range of workshops to provide their clients with a variety of self-education and self-help opportunities. From workshops and seminars, to publishing and distributing literature and hosting self-help groups, the women running the cbca and the vwhc positioned education as central to liberation. Advertising materials for the cbca’s 1976 Women’s Health Weekend told readers that “self-knowledge improves our self-concept, which is so important to our healthy functioning, and this encourages preventative health measures.”^32^ Similarly, in their 1978 analysis of the “alternative structure” of health care developed at the vwhc, Nancy Kleiber and Linda Light reffected on the importance of knowledge in the collective’s empowerment efforts: “The inseparable relationship between knowledge and the ability to control one’s health and care was central to the formation of the Health Collective. Collective members knew that it was no accident that they were consistently denied access to information about their own bodies and care. They knew that medical professionals monopolized this knowledge in order to maintain their monopoly on power. And they knew that gaining some of this knowledge was the first step toward sharing equally in this power.”^33^ In these endeavours, the vwhc and other organizations emphasized the value of the collective model in breaking down existing hierarchies, regularly insisting on “first names only” – as was the case with vwhc member “Jackie” who was introduced at the beginning of the piece – “to preserve the collective nature of their work.”^34^

Feminists debated the role of medical professionals in these new approaches to women’s health care. The goal of many women’s health centres, including the cbca and the vwhc, was to provide women with resources to become the experts of their own bodies and experiences. Self-examination, in particular, according to Kleiber and Light, provided women with “some of the awareness and the knowledge that are necessary for women to re-assume control over their own bodies.”^35^ Women’s health educators did not uniformly dismiss the role of physicians in good health care but cautioned women to find a sympathetic doctor they could trust. In their 1973 pamphlet on birth control and women’s health, Nancy Wood and Joyce Chorney of the Edmonton Family Planning Services wrote: “Some knowledge of our bodies will enable us to ask our doctors for more information. A *good* doctor doesn’t mind answering questions and has a positive attitude to women.”^36^ Some women’s health professionals actively supported and participated in this turn towards self-help in women’s health care.^37^ But, over the course of the 1970s and 1980s, many of the lay practitioners at women’s health centres increasingly questioned their relationships with the mainstream medical establishment.

## The Breast Self-Exam: Power, Prevention, and Popularization

Early literature produced and distributed by the cbca and the vwhc focused on contraceptive methods and abortion rights, but mounting concerns about cancer in the early and mid-1970s quickly made breast cancer and the bse a growing focus of the educational programs at both the vwhc and the cbca. As historian Ilana Löwy has shown, as the radical mastectomy, hysterectomy, and oophorectomy were increasingly used to treat women diagnosed with breast, uterine, and ovarian cancer, respectively, in the 1960s and 1970s, many women grappled with fears about losing their femininity and sexuality along with these parts of their bodies. These complex feelings about cancer solidified the disease and the self-exam as major topics within the women’s health movement.^38^ By the mid-1970s, the cbca and the vwhc used their newsletters to highlight resources on women and cancer and stocked their libraries with pamphlets and booklets about breast cancer, breast health, and the breast-self-exam. The cbca’s October 1974 newsletter featured a piece about the *Calgary Herald*’s review of “a book by Dr. Philip Strax, *Early Detection: Breast Cancer*” and encouraged members to learn more about breast cancer and the bse.^39^ The vwhc created and distributed booklets about the bse throughout the 1970s and 1980s, which included diagrams and detailed instructions on the method.^40^ These pamphlets similarly positioned the bse as an important preventative health practice “for early detection of breast lumps.”^41^ Throughout the 1970s, the vwhc encouraged its clients who learned how to perform the bse to tell their friends and family to learn and conduct regular self-exams and “help save a friend’s life.”^42^

**Figure 1:**
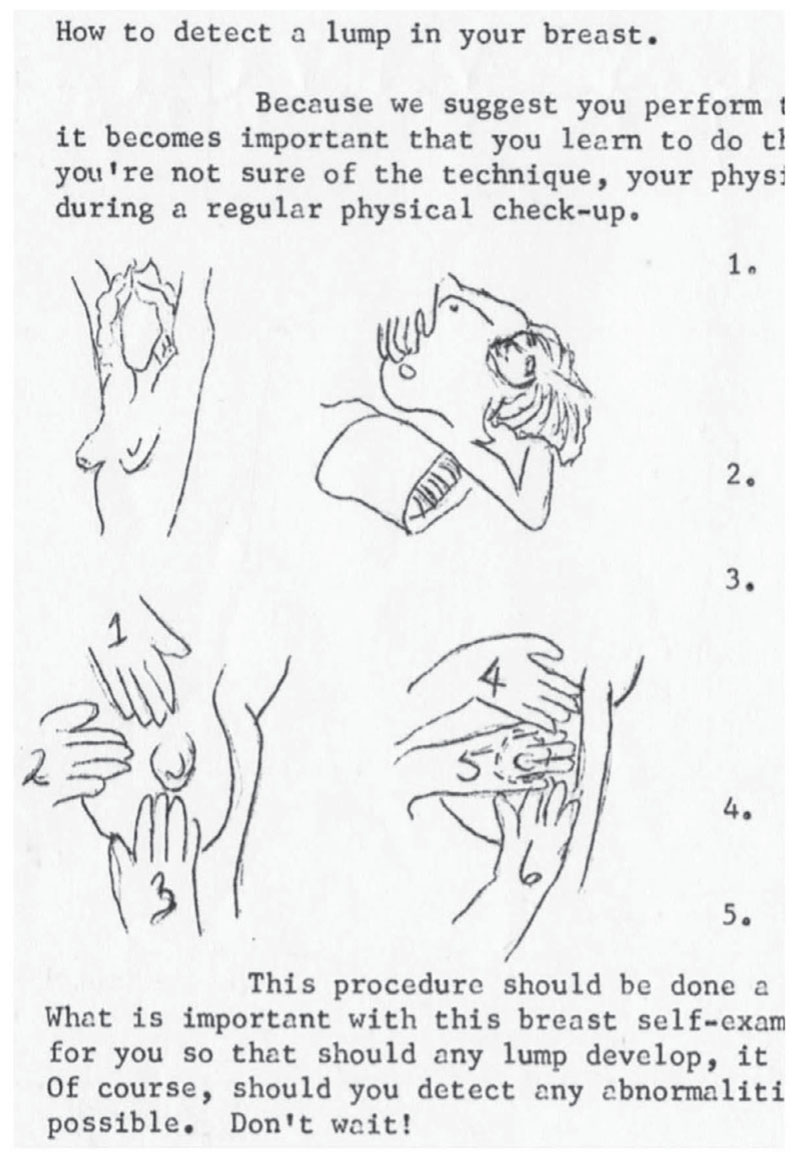
Illustrated instructions of how to conduct a bse. *Source:* Excerpt from “What about a Lump in the Breast?” *Unity* 1, no. 8 (1975): 2–3, in Lethbridge Birth Control and Information Centre, 20171104, Galt Museum and Archives, Lethbridge, Alberta.

The bse became a major feature in women’s health centres across Canada by the mid-1970s. The cbca highlighted and distributed pamphlets on the bse created by other birth control centres in Alberta. The Edmonton Family Planning Service, for example, offered instructions for a bse, complete with diagrams, in their 1973 publication *Birth Control Information Services: Health, Body, VD*, and the Lethbridge Birth Control and Information Centre published two in-depth articles about women and cancer in the April 1975 edition of their newsletter: “A Sane Look at Cancer” and “What about a Lump in the Breast” (Figure 1).^43^ The information provided by the Edmonton and Lethbridge birth control centres similarly encouraged women to “learn how to examine your breasts every month” and included hand-drawn diagrams on how to do the self-exam.^44^

As written materials created and distributed by women’s health centres across Canada increasingly featured information on breast cancer and the bse, the hands-on method of the self-exam meant that instruction had to move beyond educational literature. The bse importantly fortified women’s autonomy and expertise about their own bodies, but the variety of feelings – both physical and emotional – of individual women who conducted a bse were not easily captured in written descriptions. vwhc and cbca workers quickly realized that they had to adapt their self-education methods beyond written resources to adequately educate women about the self-exam. As early as 1973, the vwhc included bse and cse instruction in every new client-intake process. This is captured in a 1973 handout about the vwhc’s Self-Help Clinic: “If you don’t know how to examine your breasts for lumps we will teach you how, and we can show you how to see your cervix.”^45^ And a year later, in 1974, the vwhc pivoted “the orientation of its public presentations to focus on breast and cervical self-examination.”^46^ These changes solidified both bses and cses as major features in the vwhc’s educational programming. By the mid-1970s, the Calgary association similarly began hosting women’s health weekend retreats and workshops that featured entire half-day sessions on the bse.^47^

vwhc and cbca educators framed clients as the experts of their own bodies and highlighted the limits of medical professionals as they increased in-person programming on bses. In a 1976 bse group, vwhc educators explained that physicians often lacked the time for extended consultation, and many doctors saw health education on topics like the self-exam as a loss of wages because they could not charge for these services through Medicare.^48^ Beyond the lack of time that physicians had to teach the bse, educators at the cbca and vwhc also argued that women who performed a monthly breast-self-exam were familiar with their bodies and, therefore, could detect abnormalities faster than a doctor. While some physicians, like Toronto-based Dr. Leo J. Mahoney, argued that those who planned to conduct their own breast exams should consult a physician to determine what was a “normal” baseline for their breasts,^49^ educators at women’s health centres maintained that individual women were the experts on their own bodies. As Jackie, a vwhc volunteer, explained in the *Globe and Mail* article discussed at the opening of this piece, “once you’re familiar with your own breasts, you’ll be the best judge of any changes taking place.”^50^ Kleiber and Light’s report also emphasized that the breast self-exam importantly removed women’s dependence on medical professionals and, in fact, that the individual woman could “do the exam *better* herself. … She can examine her breasts more regularly and, because of the familiarity with her own healthy breasts at various stages in her cycle, she can examine them more effectively.”^51^ Indeed, by 1984, the vwhc instructional manual described the bse as “a simple, safe diagnostic tool that women have control over.”^52^

**Figure 2:**
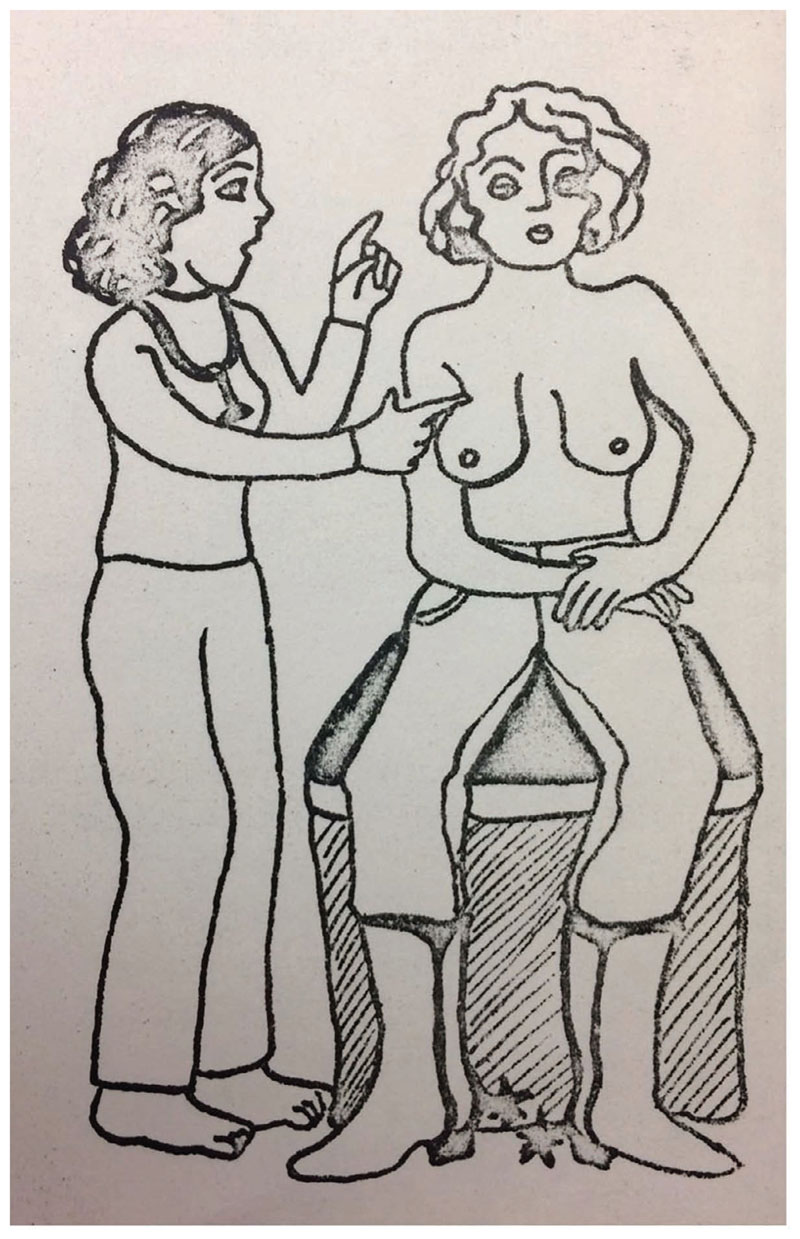
A health practitioner conducting a breast examination on a cowgirl. *Source:* cbca, Women’s Health Weekend flyer, 1976, M-7265-458, Calgary Birth Control Association Collection, Glenbow Western Research Centre, Calgary, Alberta.

As the women running the cbca and the vwhc celebrated the bse as a tool of women’s empowerment, they increasingly framed it as a way for women to take responsibility for their own health. Literature and workshop resource materials on bse from the cbca and the vwhc juxtapose dependence on medical professionals and individual women’s responsibility.^53^ In the cbca’s 1976 Women’s Health Weekend flyers and resource materials, the educators stated that “total dependence on the doctor reinforces a person’s lack of knowledge of and responsibility for her own health care.”^54^ They also argued that dependence and a lack of responsibility contribute to ill-health and told the workshop participants that “we must take control of our life and full responsibility for our bodies.”^55^ While this Women’s Health Weekend in 1976 was intended to discuss a wide range of topics, the bse was prominently featured on the flyers, which included explicit mentions of “self-examinations” along with a striking image of a lay-health practitioner instructing a topless cowgirl on how to conduct a bse (Figure 2).^56^ The vwhc similarly linked discussions of the liberative qualities of the bse with an emphasis on individual responsibility. In 1978, Kleiber and Light’s report stated that the vwhc aimed to “provide clients with knowledge and encouragement to take responsibility for themselves” and identified the self-examination, both breast and cervical, as an important tool to build up women’s liberation and responsibility: “One of the ways in which the Collective encourages women to know, accept, and take responsibility for their bodies is by teaching them breast and cervical self-exam.”^57^ At the start of 1980s, those running the cbca and the vwhc continued to discursively pair the bse with notions of women’s responsibility for their own empowerment, knowledge, and care.

Educational materials about the bse, and the concepts of liberation and responsibility that came with it, soon spread beyond the newsletters and pamphlets of women’s health centres into more mainstream publications. Leading women’s magazines, including *Chatelaine* and American titles with broad Canadian readership like *Good Housekeeping* and the *Ladies Home Journal*, discussed the bse in relation to breast cancer and encouraged women to learn about bse techniques from their doctors at their next annual physical.^58^ By the mid-1970s, popular titles celebrated the bse as an important part of women’s liberation and encouraged readers to adopt self-exam as a tool to learn about their bodies and advocate for their rights in medical spaces. Many women’s magazines featured articles about the bse, including written instructions and diagrams or photographs on how to do it. In 1974, *Redbook* included a small section on “How to examine your breasts between medical appointments” in an article called “What You Should Know about Your Breasts.”^59^ In 1975, *Chatelaine* readers learned that free leafets with diagrams on the bse were available via mail from Health and Welfare Canada.^60^ *Women’s Day* wrote that women “must take an active role in your own well-being with monthly self-examinations and not rely on the periodic examinations of your doctor” in their “Woman to Woman” segment on women’s health questions.^61^ That same year, the May issue of *Cosmopolitan* included the article “How to Examine Your Breasts,” which caught readers’ attention with the headline: “A noted gynecologist, who cares, explains how you can (and why you must) become an expert about your own breasts.” The *Cosmopolitan* article included a detailed explanation of the bse along with photographs of a woman performing the self-exam across a four-page spread.^62^ Instructions on how to perform the bse continued to be published in popular women’s magazines throughout the decade that followed.^63^ Like women’s health centres’ own material on the bse, many of the magazines encouraged women to take their health care into their own hands and learn how to conduct the bse as an important preventative health measure.

The popularization of the self-exam within mainstream women’s magazines shifted the narrative about the bse from a tool for feminist empowerment to a narrative about women’s domestic role as caregivers. Historians of medicine have explored how cancer prevention became entwined with notions of individual responsibility as biomedical research exposed links between smoking, carcinogens, and cancer. Preventative behaviour, inclusive of avoiding smoking and preforming self-exams, became part of public health messaging and popular discussions of health in late twentieth-century North America.^64^ In 1974, for example, *Women’s Day* published the article “How to Protect Your Family from Cancer,” which taught women the many ways to detect and prevent cancer in themselves, their husbands, and their children. The article covered detection and prevention for “cancers that can be cured if detected early.”^65^ While the article discussed a wide variety of cancers that afected men and women, the title of the article and the fact that it was published in *Woman’s Day* discursively positioned preventative health measures, like regularly conducting a bse, wearing sunscreen, steering clear of cigarettes, and booking your annual physical, as a woman’s responsibility. As feminist health centres continued to teach about the self-exam as an important tool of women’s empowerment, the bse, in particular, became part of a mainstream narrative about women’s role and responsibility to manage both their health and the health of their families.

By the mid-1970s, the bse had entered broader commentary about the new health-care economy in Canada. In 1974, the federal government released the Lalonde report, which underscored the importance of preventative medicine and recommended several initiatives to enhance public health.^66^ As the recommendations of the Lalonde report combined with panic about health-care spending, many Canadians were interested in how “preventative” health practices could keep their families and the economy healthy.^67^ These conversations about preventative health measures and self-help played out in the Canadian media. Reporter Stuart Lake from the *Canadian Press* published an article called “Self-help in Health Care to Be the New, Cheaper Canadian Way.” In this article, which appeared in the *Calgary Herald*, Lake described the emergence of alternative self-help health care and accessible pharmaceuticals in Canada as an exciting solution “to help cut the soaring National hospital medical bill – approaching $10 billion this year.” The article went on to tell readers that they needed to take up some of the responsibilities of their own health care. One nurse interviewed by Lake not only cautioned that people should still see doctors for their healthcare needs but also saw the beneft of self-help in preventing the health-care system from being “swamped” and health-care costs from rapidly escalating.^68^ In a similar piece, journalist Bob Cohen lamented “spiraling healthcare costs” in Canada, stating that health-care spending was projected to reach $13.8 billion by 1979.^69^ While feminists at the cbca and the vwhc framed women’s responsibility over their own health care as a liberative process, self-help health care was taken up in mainstream media to encourage individual and fiscal responsibility. Self-help practices, like the bse, which were understood as cutting significant costs when it came to expensive cancer treatments, gained popularity as they bridged the gap between empowering knowledge and an individual’s duty to cut state spending.

By the end of the 1970s and into the 1980s, the bse remained an important tool of empowerment within women’s health centres while becoming part of a more popular medical and economic discussion about preventive medicine, the health-care economy, and individual responsibility. The links to early detection of cancer and notions of individual responsibility in feminist discussions about breast self-examination were also taken up in mainstream magazines and newspapers as a solution to mounting concerns about cancer and the growing cost of the new Canadian health-care system. While some physicians claimed their expertise was required when it came to the bse, the popular discourse conceded to leave this preventative health practice in women’s hands as a fiscally responsible preventative health measure. The integration of the bse into the mainstream came with strings attached, as the feminist practice, meant to empower women, was soon positioned as women’s gendered responsibility to monitor their own health and the health of their families. The development of pelvic or cervical self-examination as a tool of feminist health activism, and the reception of cse in the popular press, was equally complicated.

## Pelvic Self-Exam: Embodied Knowledge, Contested Practice, and Medical Authority

For the cbca and the vwhc, self-examination was essential to women’s liberation from a medical system that many saw as gatekeeping and inherently patriarchal. Feminist health activists often discussed the need for breast and pelvic self-examination in tandem but within activist spaces, and, in the broader Canadian press, the two practices were markedly distinguished. While the bse increased in popularity and was taken up and celebrated as preventative medicine and a potential relief for the new Canadian health-care economy, the pelvic self-exam (pse) was seldom a topic of discussion in mainstream Canadian media and, whendiscussed, continued to be popularly positioned as a less useful, radical– and, at times, fringe – feminist act.^70^ Women’s health organizations like the vwhc and the cbca worked to counter these representations and underscore the value of vaginal, pelvic, or cervical self-exams.

**Figure 3:**
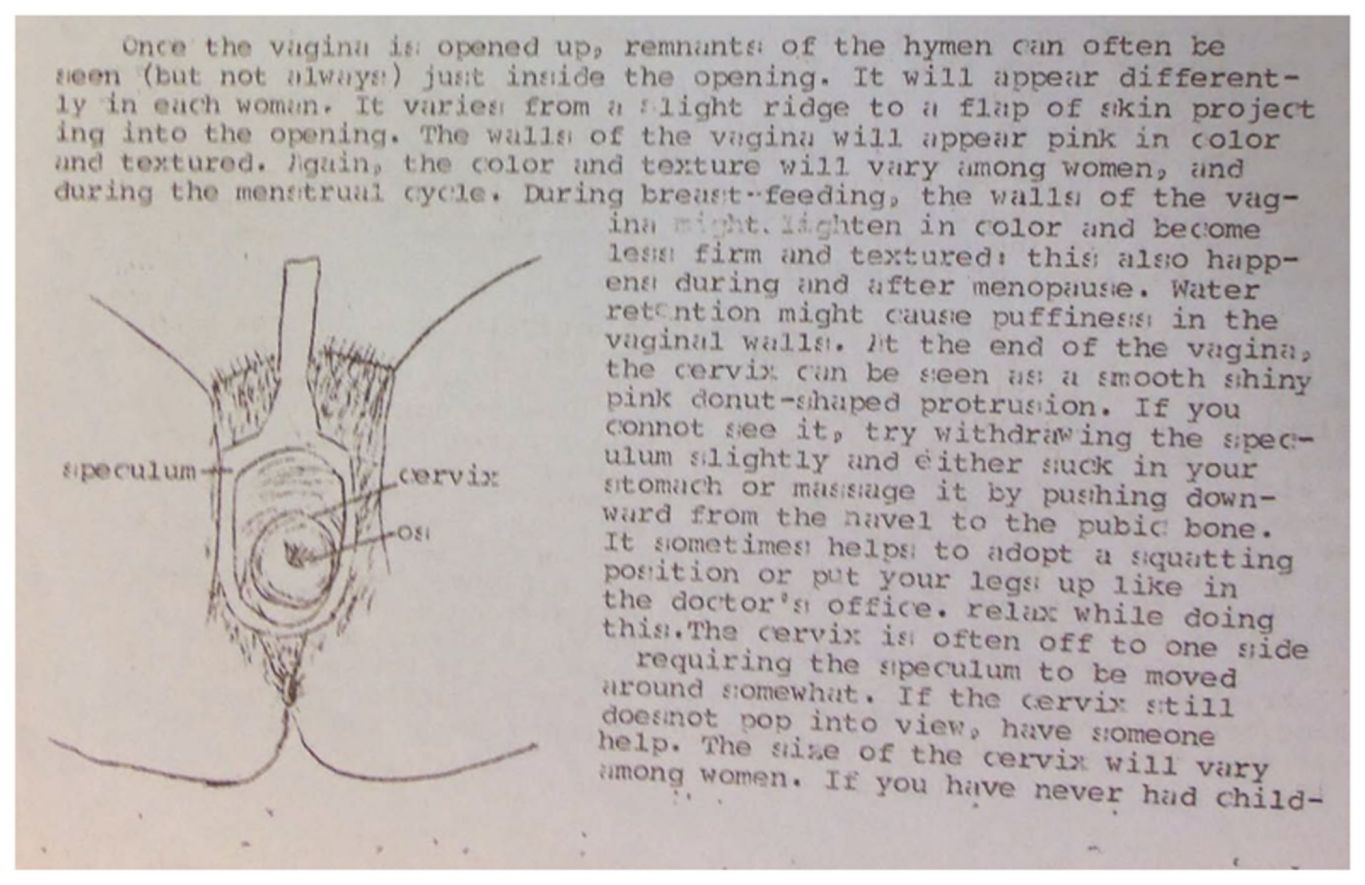
vwhc, “Information Sheets – Self Examination” (circa 1976), file 44, box 100, Canadian Women’s Movement Archives, University of Ottawa Archives.

Feminist health activists throughout North America used a variety of terms to describe what can broadly be referred to as a pelvic self-examination. Scholars of the American women’s health movement have recognized Carol Downer’s April 1971 performance of a “vaginal self-examination” at Los Angeles’ Everywoman’s Bookstore as a key event in the history of feminist health activism in the United States.^71^ Some Canadian organizations, including the vwhc, favoured and privileged the term “cervical self-exam” and the regular abbreviation of cse, a usage that reffected the value assigned to one of the central components of the practice, bringing one’s own cervix into view (Figure 3). Other organizations, including the Toronto Women’s Health Network, while using cse on occasion, relied on the term “pelvic self-examination.” This choice of language was perhaps reflective of a broadened scope of practice, including the performance of “bimanual” self-examinations, in which two fingers of the dominant hand were inserted into the vagina, while the other hand was applied to the lower abdomen to tactilely determine the position, shape, and sensitivity of the ovaries as part of establishing a broader understanding of the reproductive anatomy.^72^ On many occasions, these terms were used interchangeably across media and audiences. Here, we aim to preserve the original language used and reflected in the primary source material as much as possible.

Highlighting some of the many transnational connections of the broader North American women’s health movement, pses quickly became a mainstay of Canadian feminist health activism following the events at Everywoman’s Bookstore in the spring of 1971, and this was particularly the case for the vwhc. In the spring of 1972, a group of eleven women from the Vancouver Health Group, an immediate precursor to the vwhc, made the journey to Washington to visit the recently established Aradia Women’s Health Center in Seattle, where they were first introduced to cervical self-examination.^73^ Immediately after, according to vwhc members Nancy Kleiber and Linda Light, “they were inspired to share this experience with other Health Group members who were curious about the process, and later with women who came to the Self-Help Clinic. Cervical self-exam was thus incorporated into the Vancouver Women’s Health Collective about a year after it was first done publicly in North America in April, 1971.”^74^ Early vwhc members reported that “learning cervical self-examination” was a highlight of the collective’s first Health Group, attended by a core group of about fifteen women in the spring of 1972, with up to thirty individuals attending some sessions. cse went on to become, in their words “an extremely important component of the [vwhc’s] Women’s Self-Help Clinic.”^75^

In her study of the feminist health movement in the United States, Hannah Dudley-Shotwell notes that “gynecological self-help was contested terrain from its earliest iterations.”^76^ Looking at the early history of the vwhc, it becomes clear that the same was true in Canada as well. Surveying vwhc operations in 1978, Kleiber and Light noted that “cervical self-exam” quickly emerged as “a focus of curiosity for doctors and the press” and that the early cse practices of vwhc Health Group members, on some occasions, were “met with disgust or outright disapproval.” As a result, they argue, the vwhc did not emphasize pelvic self-examination practices in the first years of its operations.^77^ One early vwhc publication, the 1972 *Vancouver Women’s Health Booklet*, offered a vague description of the collective’s cse practices with the statement: “We have learned what we look and feel like inside.”^78^ As self-examination became – in the eyes of the vwhc, at least – a more accepted and less sensational activity in the years that followed, cse practices were more widely and explicitly advertised in the vwhc materials and in articles authored by vwhc members published in local women’s liberation publications, including *The Pedestal* and *Kinesis*.^79^ By mid-decade, vwhc activities came to firmly centre on bse and cse as the collective established a growing reputation as Canadian leaders in self-examination and other aspects of feminist health care.^80^

**Figure 4:**
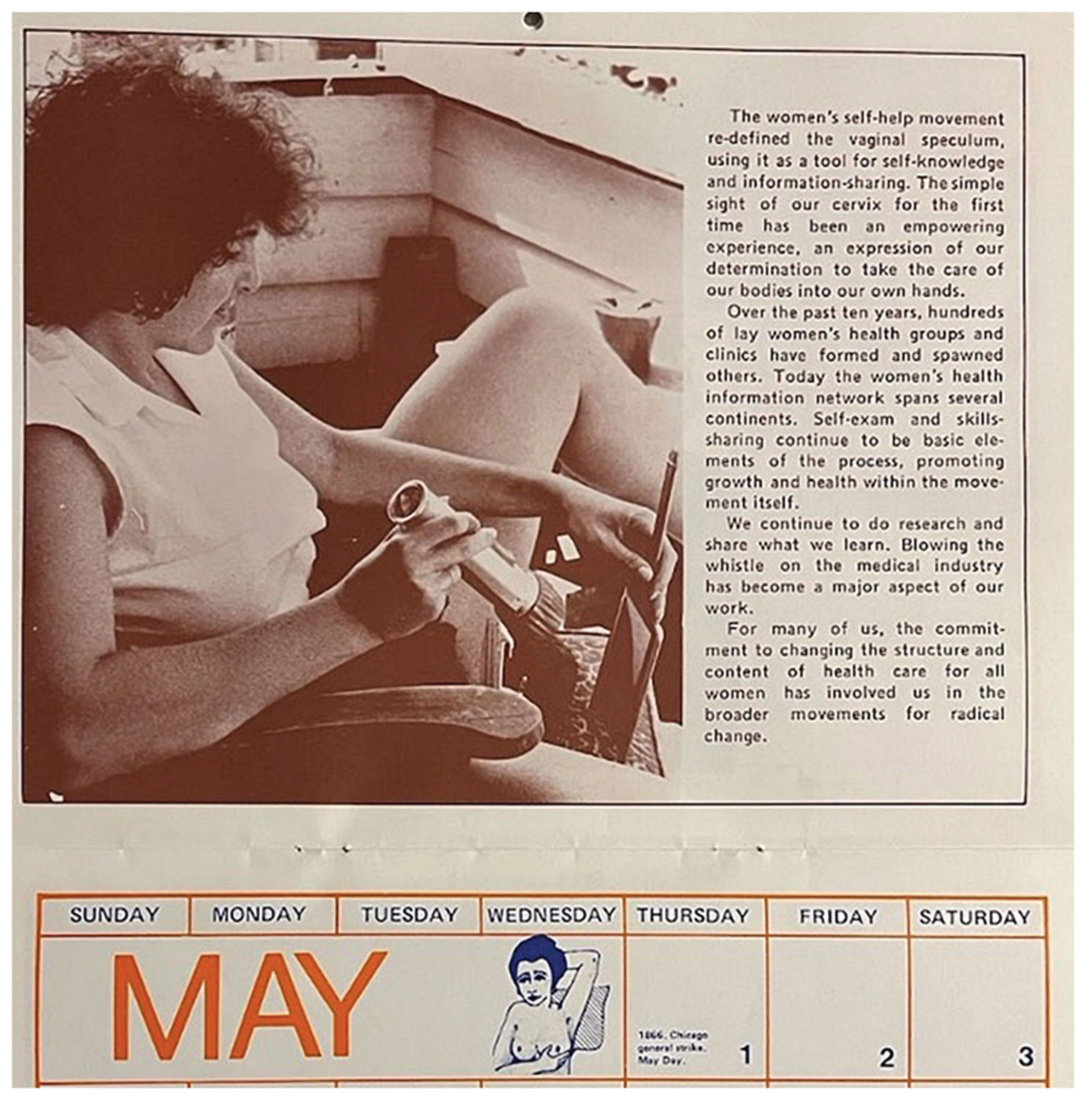
The 1980 “Women & Health” Wall Calendar produced by the vwhc featured a photograph of a woman performing a pse as the featured image for the month of May. High-lighting the significance of viewing the cervix, the accompanying text noted: “The women’s self-help movement re-defined the vaginal speculum, using it as a tool for self-knowledge and information sharing. The simple sight of our cervix for the first time has been an empowering experience, an expression of our determination to take the care of our bodies into our own hands.” This image was juxtaposed with an illustration of a bse on the adjoining calendar page. *Source:* 1980 “Women & Health” Wall Calendar, Vancouver Women’s Health Collective Archives.

The vwhc put forward a number of arguments regarding the value of cse. A three-page factsheet distributed by the collective in the mid-1970s noted that the basic intention of self-examination was “to familiarize a woman with those parts of her body which she has been denied familiarity with. … Since a doctor does not see you often enough to know what is normal for you, (s)he cannot be sensitive to the subtle changes that would enable him/her to practice preventative medicine by detecting early signs of infections, disease, and pregnancy” (Figure 4).^81^ Familiarity with one’s own body was a necessary first step in establishing a baseline that an individual could rely on in assessing their future pelvic health. Feminist health organizations also emphasized the variability and diversity of individual bodies. A 1973 pamphlet published by the Edmonton Family Planning Service included an illustration adapted from *Our Bodies, Ourselves* to accompany a discussion of self-examination, but told readers not to panic if their anatomy did not resemble the diagram.^82^ The vwhc advised clients that the appearance of the hymen, vaginal walls, and discharge varied “with each woman,” but it underscored the value of self-examination in establishing a personal baseline of pelvic health.^83^

Pelvic self-examination practices gained popularity throughout the 1970s and were often connected with broader feminist health issues, including access to contraception. The Thunder Bay, Ontario, feminist newsletter, *The Northern Woman*, for example, recommended that readers examine themselves “internally to determine the positions of both the bubic [sic] bone and the cervix” before visiting a doctor for a diaphragm fitting, suggesting that an understanding of one’s own body was foundational to effective contraceptive technique.^84^ The cbca proposed a workshop on “female anatomy and physiology,” including breast and pelvic self-examination, as part of the 1976 “Women’s Health Weekend.”^85^ Alongside an advertisement for a demonstration to be held in September 1979, the Ottawa Women’s Centre described the feminist nature of vaginal self-exam: “Pelvic examination has usually been performed by medical personnel in a clinical situation. The procedure is relatively simple, but it is helpful to watch an examination before examining yourself for the first time. It will help you feel comfortable and develop an awareness and understanding of that part of your body which we are all most alienated from. This information is not sacrosanct. It is a woman’s right.”^86^ Arguing for the demystification of “taboo” parts of the female reproductive anatomy and women’s broader control of medical knowledge, the authors of this piece positioned the use of the speculum as essential to self-exam methods. The vwhc provided cse workshop attendees with a plastic speculum at the cost of fifty cents (later eighty-five cents) and encouraged participants to take these home “to check yourself for signs of improvement or to become better acquainted with the routine signs and changes of your vagina and cervix.”^87^ Placing women’s health in women’s hands, the take-home speculum became a material symbol of feminist knowledge and resistance to patriarchal medical structures.^88^ Into the decade that followed, organizations like the vwhc continued to position the cse as a fundamental act of feminist resistance. In a February 1983 piece published in *Kinesis*, members of the vwhc described the practice of cse as “a very good way to face up to and start to change the fear and hatred of our female bodies which this male-dominated culture has taught us. For us to say that our health (and not just our illness) is our business above all, that our bodies are our own to use as we please, and that our interest in all parts of ourselves is legitimate, is a feminist statement.”^89^

Feminist health organizations also stressed the participatory and collective nature of cse. Group workshops underscored feminist arguments regarding the variability of individual bodies^90^ and provided opportunities for participants to seek help and assistance during examinations as necessary.^91^ While the performance of cse in private may have encouraged reticent participants, by the 1980s, the collective nature of cse was key to its positioning as a radical feminist act. As another example of transnational exchange, Connie Clement, one of the leading Canadian promoters of cse active in the Toronto Women’s Health Network (twhn), established in the early 1980s, reported that she first learned about the method from Lolly and Jeanne Hirsch, mother and daughter editors of the *Monthly Extract: An Irregular Periodical*, in Los Angeles in 1971. A March 1984 newsletter report on the twhn’s most recent meeting noted that “cse has always been a group activity” and one that the Network, a newer organization, was keen to adopt: “The twhn has been meeting for more than two years but until our last meeting we had not, as a group, performed cervical self-examination (cse). Because of the important place gynecological self-help has had in the women’s health movement we thought it was time to learn this technique. The opportunity to do so was met with interest and enthusiasm by the fifteen or so of us who attended the last meeting.”^92^ The vwhc, in particular, emphasized the “immediate and dramatic” impact of group cse, noting that “the sharing of both breast and cervical self-examination, which are usually very private activities, creates an emotional bond among the participants which has generally not been part of earlier collective presentations.”^93^

While individuals may not have visited a feminist health centre explicitly to learn about cse, a 1975 survey of vwhc clients indicated that approximately ten percent of respondents identified knowledge about “cervical self-examination” as the most important thing they gained from visiting the collective. In addition, some who encountered the practice at the vwhc went on to teach others how to perform self-examinations on their own. cse was a political and emotional act, with the practice of self-exam fostering feminist solidarities, as participants created – and valued – a new form of embodied knowledge based on their shared experiences.^94^ Emphasis on the feminist value of cervical self-exam persisted throughout the 1980s. The 1983 vwhc publication *A Feminist Approach to Pap Tests* continued to frame cse as “an important part of the process of women beginning to take control of and responsibility for our own bodies.”^95^ At the close of the decade, a piece appearing in the magazine of the Quebec-based birth reform organization Naissance-Renaissance positioned gynecological self-exam as “a gesture of independence and self-love” and encouraged readers to take up the practice.^96^

Despite feminists’ multi-sited efforts to promote and emphasize the value of vaginal, pelvic, or cervical self-examination, anxieties surrounding the method persisted. Within the women’s health movement, as Hannah Dudley-Shotwell and others have argued,^97^ tensions came to centre on the role of self-help gynecology as a tool to effect reform from within – or outside of – the existing medical establishment. Positioning cervical self-examination as “the central and most radical focus of the women’s health movement,” Kleiber and Light noted in 1978 that the vwhc recognized broader feminist debates surrounding the purpose of self-exam: the question, in their words, was “whether Collective members believe that they can and should work within the existing health care system to bring about change, or whether, instead, they should serve as an alternative institution, working to change the system from the outside.” While the vwhc had “no overall policy as to whether political activity should be focused on change from within or outside the health care system,” when it came to the work of collective members in educating medical and nursing students using cse, Kleiber and Light recorded that “the Collective has reached a consensus that those members who wish to participate in these activities may do so, while others continue work in other areas.^98^ Though the vwhc questioned “whether re-education of [health] professionals [was] possible,” collective members did offer bse and cse presentations to health practitioners. Medical-lay exchanges surrounding cse throughout English Canada continued into the 1980s as, on at least one occasion, feminist health activists and women interested in pse were invited to enrol in university courses intended for clinical trainees.^99^

More broadly, those who practised and promoted self-examination carefully considered and articulated their relationship to the mainstream medical establishment. Here, vwhc workers and volunteers appear to have taken a comparatively radical position, generally situating themselves as lay health workers with a distinct and woman-directed training program, who were working to promote “an alternative model for the delivery of care, which demonstrates many of the changes … necessary in the present system.” For the vwhc, these changes included an emphasis on prevention, self-help, and personal responsibility as well as the removal of sexism and professional elitism from health-care structures.^100^ In this framing, cse existed as a tool that empowered individuals to know their own bodies and pelvic health baselines, draw on individual and collective observations to “understand how to prevent, treat, and cure vaginal infections and related problems,” and “decide how to best treat [them]selves.”^101^ Other organizations, including the Ottawa Women’s Centre, positioned their self-exam work as falling within the bounds of – and valuably enriching – mainstream medical practice. Describing the centre’s work in the Ottawa-based women’s newspaper *Upstream* in 1979, Peggy Harris and Judy Lynne noted that “self-examination does not take the place of regular pelvic examinations and pap smears. However, we can and should know more about our bodies than a medical practitioner who sees us, at most, once a year, and couldn’t remember what you looked like the last time.”^102^ Here, Harris and Lynne implied that individualized knowledge of one’s own body, acquired through self-examination, could act as a valuable adjuvant to yearly medical exams. Feminist efforts to frame cse, in particular, as an aid and ally to the work of Canadian gynecologists may have been part of a conscious effort to counter broader cultural attitudes and representations that highlighted the transgressive nature of vaginal self-exam, linking the practice to menstrual extraction and the possibility of self-abortion.^103^

Throughout the 1970s and early 1980s, and in contrast to enthusiastic descriptions of the value of bse, popular North American women’s magazines were relatively silent on the practice of pelvic or cervical self-examination, with the majority of articles seeking instead to demystify the standard pelvic exam and reassure readers that they were safe in physician hands when undergoing this type of valuable preventative care.^104^ When it came to popular discussions of cervical cancer, framed as a growing health threat, *Chatelaine*, like other titles, held up the Papanicolaou or Pap smear, performed by a medically trained and licensed professional, as the gold standard screening technique. Individual articles, including a 1979 “up-to-the-minute progress report on the 10 kinds of cancer that most afflict women,” recognized the value of bse and highlighted potential early symptoms of uterine and ovarian cancers but remained silent on any possible benefits associated with examining or understanding one’s own cervix, stating plainly that “the Pap is 85 to 90 percent reliable for cervical cancer.”^105^ Articles published in leading medical journals, including the *Canadian Family Physician* and *Canadian Medical Association Journal*, similarly continued to pair the bse with the physician-performed Pap test in discussing the recommended tools of preventative practice and early cancer detection.^106^

Despite these relative silences, cervical or pelvic self-examination techniques did attract attention in the Canadian popular and medical press by the end of the decade. The *Globe and Mail*’s November 1977 profile of the vwhc, discussed in the introduction to this article, focused on both bse and cse; *Globe and Mail* readers, including physicians, however, singled out pse for comment. Ken Walker, the Ontario-based gynecologist, writing under the pseudonym W. Giford Jones, the author of the *Globe and Mail*’s recurring column, “the DOCTOR game,” for example, recognized the value of bse as “making sense” and being “sound advice in certain circumstances.” Jones pushed back, however, at length against cse, which he explicitly related to the practice of taking vaginal or pap smears for microscopic analysis.^107^ This aspect of cse – not borne out in the original *Globe and Mail* article or in the vwhc archives – was positioned as a clear transgression of the boundary between self-examination for personal knowledge and information versus diagnostic purposes, with diagnosis placed firmly in the hands and purview of the mainstream medical establishment. vwhc members Helena Summers and Marti Wendt were quick to write into the *Globe and Mail* to refute these statements and position the collective’s cse methods as preventative rather than diagnostic, stating: “We do not suggest that any women do a cervical self-examination for diagnostic purposes. In addition, pap tests and vaginal smears should only be taken by trained medical workers; no woman could ever take her own test.”^108^

Feminist efforts to respect – and perhaps to shore up – lay-medical boundaries were contextualized by medical reminders that “practice by a non-registered person” (including the application of “any apparatus or appliance”) remained an offence under provincial Medical Acts.^109^ While feminist promoters of cse heralded “the advent of the inexpensive, plastic speculum” as key to democratizing access to bodily knowledge once “thought of as accessible only to the medical profession,” physicians singled out what they cast as inappropriate use of the speculum in their opposition to self-examination practices.^110^ In the April 1978 issue of the *Canadian Family Physician*, Robin Percival-Smith, who practised family medicine at the University of British Columbia’s Student Health Service, advocated for what he described as “an educative approach to the pelvic examination.” For Percival-Smith, this new approach included consultation and conversation with the patient prior to the physical exam, the option of inserting one’s own speculum, the chance to view the cervix with the use of a mirror, and the opportunity to take home the disposable, plastic instrument following the visit, explicitly for the purposes of self-examination.^111^ Physicians responding to the article in the September issue of the journal conceded that “perhaps pelvic self-examination is desirable … within the current framework of rising health care costs and increasing practitioner workload,” but they underscored the “controversial” nature of the practice and recorded “surprise and apprehension” in reading about Percival-Smith’s techniques.^112^ Their criticisms, more specifically, highlighted the “hazard” associated with the repeated and home use of plastic and disposable specula, “instruments prone to spontaneous fracture which frequently generates injurious fragments … with many molding details and crevices which can tenaciously harbor bacteria.”^113^ Here, these practitioners centred what they framed as the broader health risks associated with placing the tools of “women’s health in women’s hands.” Medical resistance to self-examination techniques, particularly in terms of what some physicians perceived as lay incursions into medical practice and challenges to physician expertise, continued alongside an atmosphere of antagonism between select practitioners and feminist health activists (including the feminist press) into the 1980s.^114^

## Conclusion

The 1970s was a transformative decade for both women’s liberation and the development of Canadian Medicare. As individual provinces developed and implemented health-care policies, feminist activists quickly recognized reproductive and sexual health as an area of inadequate service provision. To address these inequities, local activists created alternative models of care and service provision in their communities, positioning self-help as an integral tool of feminist liberation. While organizations like the cbca and the vwhc served a diverse clientele, broader feminist concepts of women’s universal experiences sometimes limited the perspectives within these centres and the women’s health movement to white, urban, and middle-class health priorities. Despite these limitations, for many who visited health centres and collectives, the self-examination became a significant tool in placing women’s health in women’s hands, developing collective knowledge, and ensuring more comprehensive attention to breast and pelvic health.

Shifting narratives about individual responsibility for health and the balance between lay and medical authority intersected to shape conversations surrounding self-examination in the 1970s and 1980s. While feminist health activists often jointly discussed breast and pelvic self-examination, Canadian commentators distinguished between the two practices. The bse was taken up as an important preventative health measure in popular newspapers and magazines, entrenching the practice in two distinct popular discourses: fighting medical patriarchy and curbing the growing health-care economy. Both narratives positioned the bse, and health prevention more broadly, as part of women’s responsibility for individual, familial, and societal health and well-being, an emphasis that continues into the twenty-first century.^115^ pse or cse, in contrast, remained on the margins of mainstream discussions of cervical cancer risk and, when discussed, was popularly positioned as a fringe feminist act. While health activists and organizers lauded the transformational value of cse in allowing women to fully know their own bodies, the radical nature of the pse, which involved the appropriation of medical tools like the speculum, and the subversive possibility of moving beyond observation to diagnosis, attracted pushback from physicians for its perceived incursions into medical authority. Nevertheless, and despite the limitations of self-help models, feminist health activists, including those afliated with the vwhc and the cbca, relied on self-examination methods to foster and demonstrate women’s autonomy over their own bodies and to fuel the development of alternative, collective systems of care and broader efforts to reform the Canadian medical establishment.

## Footnotes

1 Elizabeth Whynot, “Still Standing,” in *Personal and Political: Stories from the Women’s Health Movement, 1960–2010*, ed. Lorraine Greaves (Toronto: Second Story Press, 2018), 70.

2 Anne Roberts, “Clinic Teaches Women How to Examine Selves for Symptoms of Disease,” *Globe and Mail*, 10 November 1977, F5.

3 Ibid., F5.

4 Nancy Kleiber and Linda Light, *Caring for Ourselves: An Alternative Structure for Health Care* (Vancouver: Vancouver Women’s Health Collective [VWHC], 1978), 22.

5 Historians of the body, including Barbara Duden, have demonstrated the value of individual narratives of embodied experience in shaping medical care, as personal descriptions of illness and health, articulated based on variations from what was “normal” for each individual, were regularly the most important factor in determining diagnosis and treatment throughout the early modern period. Barbara Duden, *The Woman beneath the Skin: A Doctor’s Patients in Eighteenth-Century Germany* (Cambridge, MA: Harvard University Press, 1991).

6 Jean Donnison, *Midwives and Medical Men: A History of Inter-Professional Rivalries and Women’s Rights* (New York: Shocken Books, 1977); Ann Oakley, *The Captured Womb: A History of the Medical Care of Pregnant Women* (New York: Basil Blackwell, 1984); Judith Walzer Leavitt, *Brought to Bed: Childbearing in America, 1750 to 1950* (New York: Oxford University Press, 1986); Leslie J. Reagan, *When Abortion Was a Crime: Women, Medicine, and Law in the United States, 1867–1973* (Berkeley: University of California Press, 1997).

7 Cynthia Comacchio, *Nations Are Built of Babies: Saving Ontario’s Mothers and Children, 1900–1940* (Montreal and Kingston: McGill-Queen’s University Press, 1993); Wendy Mitchinson, *Giving Birth in Canada, 1900–1950* (Toronto: University of Toronto Press, 2002); Angus McLaren and Arlene Tigar McLaren, *The Bedroom and the State: The Changing Practices and Politics of Contraception and Abortion in Canada, 1880–1980* (Toronto: McClelland & Stewart, 1986); Christabelle Sethna et al., “Choice, Interrupted: Travel and Inequality of Access to Abortion Services in the 1960s,” *Labour/Le Travail* 71 (2013): 29–48; Shannon Stettner, *Without Apology: Writings on Abortion in Canada* (Edmonton: Athabasca University Press, 2016); Erika Dyck and Maureen Lux, *Challenging Choices: Canada’s Population Control in the 1960s* (Montreal and Kingston: McGill-Queen’s University Press, 2020).

8 Wendy Mitchinson, *The Nature of Their Bodies: Women and Their Doctors in Victorian Canada* (Toronto: University of Toronto Press, 1991); Wendy Mitchinson, *Body Failure: Medical Views of Women, 1900–1950* (Toronto: University of Toronto Press, 2013).

9 Patricia Jasen, “From the ‘Silent Killer’ to the ‘Whispering Disease’: Ovarian Cancer and the Uses of Metaphor,” *Medical History* 53, no. 4 (2009): 489–512; Patricia Jasen, “Breast Cancer and the Language of Risk, 1750–1950,” *Social History of Medicine* 15, no. 1 (2002): 17–43; Mandy Hadenko, *“Talking about Down There”: The Development of a Public Discussion of Cervical Cancer in the Twentieth Century* (PhD diss., York University, 2014); Jennifer Fraser, “Seizing the Means of Reproduction? Canada, Cancer Screening, and the Colonial History of the Cytopipette,” *Canadian Bulletin of Medical History* 38, no. 1 (2021): 128–76.

10 Susan Smith, *Sick and Tired of Being Sick and Tired: Black Women’s Health Activism in America, 1890–1950* (Philadelphia: University of Pennsylvania Press, 1995); Sandra Morgen, *Into Our Own Hands: The Women’s Health Movement in the United States, 1969–1990* (New Brunswick: Rutgers University Press, 2002); Wendy Kline, *Bodies of Knowledge: Sexuality, Reproduction, and Women’s Health in the Second Wave* (Chicago: University of Chicago Press, 2010); Jennifer Nelson, *More Than Medicine: A History of the Feminist Women’s Health Movement* (New York: New York University Press, 2015); Hannah Dudley-Shotwell, *Revolutionizing Women’s Healthcare: The Feminist Self-Help Movement in America* (New Brunswick, NJ: Rutgers University Press, 2020).

11 Kathy Davis, *The Making of* Our Bodies, Ourselves: *How Feminism Travels across Borders* (Durham, NC: Duke University Press, 2008).

12 For more on grassroots women’s health projects during this period, see Margaret MacDonald, *At Work in the Field of Birth: Midwifery Narratives of Nature, Tradition, and Home* (Nashville: Vanderbilt University Press, 2008); Megan J. Davies, “Women Unafraid of Blood: Kootenay Community Midwives, 1970–1990,” *BC Studies* 183 (2014): 11–36; Jenny Ellison, *Being Fat: Women, Weight, and Feminist Activism in Canada* (Toronto: University of Toronto Press, 2020).

13 Christabelle Sethna, “The Evolution of the *Birth Control Handbook:* From Student Peer-Education Manual to Feminist Self-Empowerment Text, 1968–1975,” *Canadian Bulletin of Medical History* 23, no. 1 (2006): 89–118; Christabelle Sethna, “The University of Toronto Health Service, Oral Contraception, and Student Demand for Birth Control, 1960–1970,” *Historical Studies in Education* 17, no. 2 (2005): 265–92; Megan Blair, “‘Babies Needn’t Follow’: Birth Control and Abortion Policy and Activism at the University of Waterloo and Waterloo Lutheran University, 1965–74,” *Canadian Bulletin of Medical History* 37, no. 1 (2020): 88–118.

14 Greaves, *Personal and Political*.

15 Megan Davies, “Mapping ‘Region’ in Canadian Medical History: The Case of British Columbia,” *Canadian Bulletin of Medical History* 17, no. 1 (2000): 73–92; Esyllt Jones, *Influenza 1918: Disease, Death, and Struggle in Winnipeg* (Toronto: University of Toronto Press, 2007); Lianne McTavish, “Abortion in New Brunswick,” *Acadiensis* 44, no. 2 (2015): 107–30; Catherine Carstairs, *The Smile Gap: A History of Oral Health and Social Inequality* (Montreal and Kingston: McGill-Queen’s University Press, 2022).

16 Monika Baar, “Seeking Inclusion through Redefning Expertise: The Changing Spatial Contours of Disability Activism in the Long 1970s,” *European Review of History* 29, no. 3 (2022): 452–68; Eve Worth, “Women, Education and Social Mobility in Britain during the Long 1970s,” *Cultural and Social History* 16, no. 1 (2019): 67–83; Ian M. Baldwin, “Rethinking the ‘Era of Limits’: Equitable Housing, Gay Liberation, and the Opening of the American Family in Greater Los Angeles during the Long 1970s,” *California History* 91, no. 3 (2014): 42–59.

17 “Progress Report,” 10 April 1972; “Progress Report,” 1 June 1972, M-7265-185, Calgary Birth Control Association Collection (CBCA Collection), Glenbow Western Research Centre, Calgary, AB.

18 Kleiber and Light, *Caring for Ourselves*, 22; VWHC, “About Us,” https://womenshealthcollective.ca/about-us/

19 See, for example, VWHC, “Who We Are … Women’s Self-Help Clinic,” c. 1975–6, M-7265-457, CBCA Collection.

20 Gregory P. Marchildon, “Canadian Medicare: Why History Matters,” in *Making Medicare: New Perspectives on the History of Medicare in Canada*, ed. Gregory P. Marchildon (Toronto: University of Toronto Press, 2012), 3, 6–7. For more on the history of Medicare, see Heather MacDougall, “Into Thin Air: Making National Health Policy, 1939–45,” in Marchildon, *Making Medicare*, 41–70; Esyllt Jones, James Hanley, and Delia Gavrus, eds., *Medicare’s Histories: Origins, Omissions and Opportunities in Canada* (Winnipeg: University of Manitoba Press, 2022).

21 Karissa Patton and Emily Kaliel, “Building Community and Transforming Knowledge: Histories of Women’s Health Practitioners and Community-Based Health Services in 20th-Century Alberta, Canada,” *Canadian Bulletin of Medical History* 37, no. 2 (2020): 427–60.

22 McTavish, “Abortion in New Brunswick,” 107–30; Patton and Kaliel, “Building Community, Transforming Knowledge,” 427–60.

23 Davis, *Making of* Our Bodies, 21; Kline, *Bodies of Knowledge*; Matthew J. Sobnosky, “Experience, Testimony, and the Women’s Health Movement,” *Women’s Studies in Communication* 36, no. 3 (2013): 217–18.

24 Benita Roth, *Separate Roads to Feminism: Black, Chicana, and White Feminist Movements in America’s Second Wave* (Cambridge, UK: Cambridge University Press, 2004); Wendy Kline, “The Making of *Our Bodies, Ourselves*: Rethinking Women’s Health and Second-Wave Feminism,” in *Feminist Coalitions: Historical Perspectives on Second Wave Feminism in the United States*, ed. Stephanie Gilmore and Sara Evans (Urbana: University of Illinois Press, 2008), 63–83; Linda Nicholson, *The Second Wave: A Reader in Feminist Theory* (New York: Routledge, 1997), 3–4.

25 Karissa Patton, “Con(tra)cepts of Care: Southern Alberta Birth Control Centres and Reproductive Healthcare, 1969–1979” (PhD diss., University of Saskatchewan, 2021); Brenda Jones, “Health Advocates Help Women: Volunteers Try to Get Rid of Confusion That Arises from Cultural Diferences,” *Vancouver Echo*, 3 March 1999.

26 Kline, “Making of *Our Bodies, Ourselves*,” 63–83.

27 Roth, *Separate Roads to Feminism*; Kline, “Making of *Our Bodies, Ourselves*,” 63–83; Nicholson, *Second Wave*, 3–4; Patton and Kaliel, “Building Community, Transforming Knowledge,” 427–60.

28 See “Conferences: Self-Help ‘72 Calgary,” 1972, M-7265-69, CBCA Collection.

29 VWHC, *The Concept of Self-Help* (Vancouver: VWHC, c. 1977), 1.

30 Kleiber and Light, *Caring for Ourselves*, 16; see, for example, “Resource Materials: Miscellaneous Pamphlets on Women’s Health, Birth Control, etc. ca.1960s–1990s,” c. 1967–90, M-7265-355, CBCA Collection.

31 Sobnosky, “Experience, Testimony,” 218.

32 “Calgary Birth Control Association Special Project to P.S.S.,” c. 1975–6, M-7265-458; “Women and Health” *CBCA Newsletter*, November 1974, 5, M-7265-240, CBCA Collection.

33 Kleiber and Light, *Caring for Ourselves*, 16.

34 Roberts, “Clinic Teaches Women,” F5.

35 Kleiber and Light, *Caring for Ourselves*, 58.

36 Nancy Wood and Joyce Chorney, *Birth Control Information Services: Health, Body, VD*, Edmonton Family Planning Service, September 1973, M-7265-355, CBCA Collection (emphasis in the original).

37 “CBCA ‘Prevention’ Defended by Doctor,” *Calgary Herald*, 14 February 1975, 26, M-7265-170, CBCA Collection; “Doctors at the Self-help Clinic,” in Kleiber and Light, *Caring for Ourselves*, 133–42.

38 Ilana Löwy, *Preventive Strikes: Women, Precancer, and Prophylactic Surgery* (Baltimore: Johns Hopkins University Press, 2010), 11, 199–201.

39 *CBCA Newsletter*, October 1974, 4, M-7265-240, CBCA Collection.

40 VWHC, *Breast Self-Exam* (Vancouver: VWHC, c. 1983–4).

41 VWHC, *Breast Health*, Self-Help Workshop flyers (Vancouver: VWHC, c. 1980s).

42 Kleiber and Light, *Caring for Ourselves*, 116.

43 Wood and Chorney, *Birth Control Information Services*; “A Sane Look at Cancer: “What about a Lump in the Breast?” *Unity* 1, no. 8 (1975): 2–3, Lethbridge Birth Control and Information Centre, 1973–1975, Galt Museum and Archives, Lethbridge, AB.

44 “Sane Look at Cancer,” 2; Wood and Chorney, *Birth Control Information Services*.

45 VWHC, *The Women’s Self-Help Clinic* (Vancouver: VWHC, 1973), 2.

46 Kleiber and Light, *Caring for Ourselves*, 22.

47 VWHC, *Women’s Health Series* flyer (Vancouver: VWHC, c. 1975); “Calgary Birth Control Association Special Project to P.S.S.,” c. 1975–6, M-7265-458, CBCA Collection; “Women and Health,” *CBCA Newsletter*, November 1974, 5, M-7265-240, CBCA Collection; “Description of the Well Woman Seminar,” 1978, M-7265-457, CBCA Collection.

48 Kleiber and Light, *Caring for Ourselves*, 167.

49 “Self-examination for Breast Cancer Won’t Work If Woman Doesn’t Know What’s Normal,” *Globe and Mail*, 11 November 1976, F5.

50 Roberts, “Clinic Teaches Women,” F5.

51 Kleiber and Light, *Caring for Ourselves*, 116 (emphasis in original).

52 VWHC, “Breast Self-Exam,” *Facilitating Self-Help Workshops* (Vancouver: VWHC, 1984).

53 “Description of the Well Woman Seminar,” 1978, M-7265-457, CBCA Collection; Kleiber and Light, *Caring for Ourselves*, 11, 16.

54 “Calgary Birth Control Association Special Project to P.S.S.,” c. 1976, M-7265-458, CBCA Collection.

55 “Frances Wren on Women, Health, Medicine, and Doctors,” c. 1976, M-7265-458, CBCA Collection.

56 “Calgary Birth Control Association Special Project to P.S.S.,” c. 1976, M-7265-458, CBCA Collection.

57 Kleiber and Light, *Caring for Ourselves*, 16, 11.

58 Timothy H. Mulligan et al., “The Physical Examination: What You Have the Right to Expect,” *Good Housekeeping*, April 1976, 189; David R. Zimmerman, “Medicine Today,” *Ladies’ Home Journal*, February 1974, 26; Elizabeth Morgan, “Your Body,” *Cosmopolitan*, August 1978, 24.

59 “What you should know about your breasts,” *Redbook*, January 1974, 108, 110.

60 Bonnie Cornell, “Of Consuming Interest,” *Chatelaine*, October 1975, 37, 40.

61 Lucienne T. Lanson, “Women to Woman: Over Fifty Answers to the Most Intimate Questions Women Ask Their Doctors,” *Woman’s Day*, February 1975, 60–1, 79–80, 82, 84.

62 Selig Neubardt, “How to Examine Your Breasts.” *Cosmopolitan*, May 1975, 90, 92, 94, 96.

63 Marcia Storch and Loretta May, “The Complete Breast Book,” *Good Housekeeping*, February 1979, 75, 78, 76–7, 81–3; June Rogers, “10 Do-It-Yourself Medical Tests,” *Chatelaine*, June 1985, 12, 20.

64 Löwy, *Preventive Strikes*, 11; Carsten Timmermann, *A History of Lung Cancer: The Recalcitrant Disease* (London: Palgrave Macmillan, 2014), 151–5.

65 Jane E. Brody, “How to Protect Your Family from Cancer,” *Woman’s Day*, November 1974, 58, 60, 108, 110, 113–14.

66 Marc Lalonde, National Health and Welfare Canada, A New Perspective on the Health of Canadians: A Working Document, April 1974, reprinted 1981, https://www.phac-aspc.gc.ca/ph-sp/pdf/perspect-eng.pdf.

67 Helen Vanderberg et al., “The History of Saskatchewan’s Public Health Association Policy Initiatives, 1954–86: A Regional Comparison for Preventative Health Policy Work in Canada,” *Canadian Bulletin of Medical History* 38, no. 1 (2021): 115–16.

68 Stuart Lake, “Self-help in Health Care to Be the New, Cheaper Canadian Way,” *Calgary Herald*, 24 February 1978, D20, clipping, M-7265-457, CBCA Collection.

69 Bob Cohen, “‘Self-care’ Nothing New, but Slowing Health Costs,” 24 February 1978, C9, clipping, M-7265-457, CBCA Collection.

70 Letter from E. Odette Hagen (MD) to CBCA c/o Anne Eaton, 9 February 1978, M-7265-457, CBCA Collection.

71 Michelle Murphy, “Immodest Witnessing: The Epistemology of Vaginal Self-Examination in the U.S. Feminist Self-Help Movement,” *Feminist Studies* 30, no. 1 (2004): 115–47; Morgen, *Into Our Own Hands*; Kline, *Bodies of Knowledge*; Dudley-Shotwell, *Revolutionizing Women’s Healthcare*.

72 “NOT the Oldest Profession: Two Women Talk About Their Experiences as Professional Patients,” *Healthsharing*, 6, no. 2 (Spring 1985): 12.

73 Nelson, *More Than Medicine*, 91–122.

74 Kleiber and Light, *Caring for Ourselves*, 57.

75 Ibid., 97.

76 Dudley-Shotwell, *Revolutionizing Women’s Healthcare*, 11.

77 Kleiber and Light, *Caring for Ourselves*, 58.

78 VWHC, *A Vancouver Women’s Health Booklet* (Vancouver: Press Gang, 1972), 83.

79 VWHC, *Health Phone Line, Health Groups, Women’s Self-Help Clinic*, information leafet (Vancouver: VWHC, October 1973); VWHC, “In Sickness and in Health,” *The Pedestal: The Vancouver Women’s Liberation Newspaper* 5, no. 5 (1973): 2–3; “Resource Bank: Medical Resources,” *Kinesis: Vancouver Status of Women* 4, no. 31 (1974): 2b; “North Shore,” *Kinesis* 5, no. 50 (1975): 3.

80 Kleiber and Light, *Caring for Ourselves*, 22, 58.

81 vwhc, “Information Sheets – Self Examination,” c. 1976, fle 44, box 100, Canadian Women’s Movement Archives (CWMA), University of Ottawa Archives.

82 Wood and Chorney, *Birth Control Information Services*, 30–1.

83 VWHC, “Information Sheets – Self Examination,” c. 1976.

84 “Birth Control Methods,” *The Northern Woman* 13 (1974): 5. Pelvic self-examination was often connected to diaphragm ftting. See also Jeanette Frost, “The Diaphragm Fitters Collective,” in Greaves, *Personal and Political*, 74–6.

85 “Calgary Birth Control Association Special Project to P.S.S.,” c. 1976, M-7265-458, CBCA Collection.

86 Peggy Harris and Judy Lynne, “Well-Women: Vaginal Self-Exam,” *Upstream* 3, no. 9 (1979): 9.

87 VWHC, “Who We Are … Women’s Self-Help Clinic,” c. 1975–6.

88 See, for example, the comic-style cartoon of Wonder Woman holding a speculum and stating: “With my Speculum, I am Strong! I can Fight!” Published on the Cover of *Sister*, the Los Angeles Women’s Center Newspaper in July 1973.

89 Robyn Barnett and Rebecca Fox, “Cervical Self-examination,” *Kinesis*, February 1983, 2.

90 *Toronto Women’s Health Network Newsletter*, March 1984, CWMA.

91 The VWHC, for example, encouraged women to seek help if they faced diffculties in opening the speculum or bringing the cervix into view. VWHC, “Information Sheets – Self Examination,” c. 1976.

92 *Toronto Women’s Health Network Newsletter*, March 1984, CWMA.

93 Kleiber and Light, *Caring for Ourselves*, 108–9.

94 Ibid., 128, 131.

95 Robin Barnett and Rebecca Fox, *A Feminist Approach to Pap Tests* (Vancouver: VWHC, 1983), 8.

96 “L’auto-examen gynecologique” was described as “un geste d’independance et d’amour de soi-meme.” Shirley Rivet, “L’auto-examen gynecologique,” *L’Une a L’autre: La Revue de Naissance-Renaissance* 4, no. 3 (1987): 18.

97 Dudley-Shotwell, *Revolutionizing Women’s Healthcare*, 28.

98 Kleiber and Light, *Caring for Ourselves*, 56, 110.

99 *Toronto Women’s Health Network Newsletter*, April 1987, CWMA.

100 VWHC, “Who We Are … Women’s Self-Help Clinic,” c. 1975–6; Kleiber and Light, *Caring for Ourselves*, 110.

101 Robin Barnett, “Inside Women,” *Kinesis*, September 1984, 8.

102 Harris and Lynne, “Well-Women,” 9.

103 Connie Clement, “The Case for Lay Abortion: Learning from Midwifery,” *Healthsharing*, 4, no. 4 (Winter 1983): 14.

104 Lorna and Philip Sarrel, “Understanding the Pelvic Exam,” *Redbook*, January 1982, 36, 38.

105 See, for example, June Engel, “Cancer & Women: An Up-to-the-Minute Progress Report,” *Chatelaine*, August 1979, 27, 84, 86, 88–90.

106 A.J. Phillips and M.E. Brennan, “Reactions of Canadian Women to Pap Tests and Breast Self-Examination,” *Canadian Family Physician* 22, no. 1261 (1976): 71–4; Renaldo N. Battista et al., “Patterns of Preventive Practice,” *Canadian Medical Association Journal* 132, no. 9 (1 May 1985): 1013-15.

107 W. Giford Jones, “The DOCTOR Game: Self-examination Clinics Need More Than Good Intentions,” *Globe and Mail*, 8 December 1977, F5.

108 Helena Summers and Marti Wendt, “Self-help Clinic Teaches Preventive Care,” *Globe and Mail*, 30 January 1978, 7.

109 The language here was included in the Alberta Medical Profession Act in 1975. Letter from E. Odette Hagen (MD) to CBCA c/o Anne Eaton, 9 February 1978.

110 Barnett, “Inside Women,” 8.

111 Robin Percival-Smith, “An Educative Approach to the Pelvic Examination in Young Women,” *Canadian Family Physician* 24 (1978): 357–61.

112 The authors of this letter were physicians employed by the federal Bureau of Medical Devices, and their letter concluded with the note: “The views expressed herein are solely those of the authors and do not necessarily reflect existing or forthcoming policies of the Department of National Health and Welfare.” R.W. Campbell, M.T. Cooper, and P. Blais, “Should Patients Do Own Pelvic Exams?” *Canadian Family Physician* 24 (1978): 818–19.

113 Ibid., 818–19.

114 Dr. A[rnold] Soichet of Mission, BC, for example, spoke out against what he perceived as *Healthsharing*’s “anti-medical bias” in the magazine’s winter 1984 issue. A. Soichet, “Letter to the Editor – Anti-Medical Bias,” *Healthsharing* 6, no. 1 (Winter 1984): 23.

115 Stephanie C. Roy, “‘Taking Charge of Your Health’: Discourses of Responsibility in English-Canadian Women’s Magazines,” *Sociology of Health & Illness* 30, no. 3 (2008): 463–77.

